# The Case of Dr. Imlach

**Published:** 1901-03-09

**Authors:** 


### The Hospital

A Journal of

The Medical Sciences and Hospital Administration.

Vol. XXIX.—No. 754.] Edited by Sir Henry Burdett, K.C.B., and Solomon C. Smith, M.D., M.R.C.P. [March 9, 1901.

### The Case of Dr. Imlach.

The public mind of Liverpool has recently been much disquieted by the consideration of questions of no small importance, alike to the public itself and to the profession, which have been brought into new prominence by the re-opening of what is described as the "case of Dr. Imlach," although, in reality, it is one of a very much wider scope than the name of any individual actor in it would at first sight imply. It goes back to the year 1885, at which time the practice pursued at a local hospital for women, called the "Shaw Street Hospital," had excited a good deal of comment on account of an alleged undue frequency of serious abdominal operations within its walls. The operations chiefly criticised or objected to were apparently of two classes—the removal of the ovaries for one, and the opening of the abdominal cavity in cases of pelvic hæmorrhage from tubal pregnancy for another—and it was said or believed that, in respect of both, Dr. Imlach, one of the physicians, was the officer chiefly responsible for the supposed excess. There does not appear to have been even any profession of precise knowledge on the part of any of the critics of the exact condition and circumstances of the patients upon whom the operations supposed to have been superfluous had been performed, and we do not hear that there had been any complaints from patients or from their friends with regard to the manner in which they had been treated. There was, in short, nothing more than a diffused suspicion that, in the hospital in question, operative treatment had been carried to excess.

In this disturbed condition of the atmosphere there was held, on February 4th, 1886, an ordinary meeting of the Liverpool Medical Institution—a society described in its own laws as constituted "for the cultivation of medicine, surgery, and the collateral branches of science exclusively, together with the maintenance of a library." The business before the meeting was to hear and discuss the history of a case of renal calculus; but, as if from the result of some intimation privately given to members likely to be interested, there was a somewhat full attendance. In the course of the evening, without any notice on the agenda, and without any other form of public announcement, a resolution was moved, seconded, and carried by a considerable majority, "That, in view of the large and increasing number of cases of abdominal section in the Hospital for Women in this city, as shown in their annual medical reports for 1884 and 1885, this meeting is of opinion that a select committee should be appointed for the purpose of investigating the grave questions of practice and ethics involved in the performance of the operations, and to report to a future meeting." Objections of rule and form to the whole procedure were promptly raised in various quarters, but were defeated. The committee held a number of meetings, and concluded its labours by a report bearing considerable resemblance to the summing up of Mr. Justice Stareleigh in the historical case of Bardell *versus* Pickwick. It was perfectly clear that the committee had not in the least degree grappled with the questions really at issue, and that they had not had before them a scintilla of evidence to show either that any single operation had been injurious or unnecessary, or that the consent of the patient to submit to it had been obtained by means of representations not warranted by the facts of the case. They note that, out of 106 patients subjected to removal of the ovaries and tubes, nine died; but they adduced nothing which would support the belief that these deaths were other than unavoidable. They made certain recommendations with which everybody would agree, except in so for as they might be taken to indicate that the spirit of them had previously been neglected; and, in fact, they extricated themselves from a painful and untenable position in a manner not without a certain dexterity of non-committal. Unfortunately, the dominant party in the institution thought fit to publish this report, a report which could only be regarded as having been made to a scientific society for its own guidance on a scientific question, in the non-medical local press, with the inevitable result that the whole controversy became widely known and discussed in the city and neighbourhood, more especially among the lay governors of the hospital concerned. The medical staff of the hospital were subject to annual re-election, which, as a rule, was a matter of course; but the first result of the publication of the report was that Dr. Imlach was not re-elected at the next general meeting, but was deprived of his appointment and of all the opportunities of study and advancement which it might imply, with the result that his practice was very seriously diminished by the slur supposed to be cast upon him. During the fifteen years which have elapsed the principles on which he acted, and for which he contended, have come to be recognised as sound; and the only form of operation which the committee ventured to condemn, that for pelvic hæmatocele, it would now be regarded as malpractice to neglect. In these circumstances Dr. Imlach and his friends think it is time that something was done to remove the stigma under which he has laboured for so long; and an active endeavour is being made to procure at the hands of the institution the deletion of a record which ought never to have been placed upon its minutes. Such deletion, if carried, would naturally and properly be regarded by the public as an admission that Dr. Imlach had in the first instance been wrongfully treated; and this admission seems to us to be clearly his due. We are informed that the opposition to the proposal rests upon the assumption that, if it were adopted, some reflection would be cast upon the honour of the original committee. We do not agree with this opinion. All men, even all committee-men, are liable to err; and, especially when error has inflicted injury upon others, the only course consistent with honour is to make such amends as the circumstances may admit.

